# New Trends in Esophageal Cancer Management

**DOI:** 10.3390/cancers13123030

**Published:** 2021-06-17

**Authors:** Caroline Gronnier, Denis Collet

**Affiliations:** 1 Eso-Gastric Surgery Unit, Department of Digestive Surgery, Magellan Center, Bordeaux University Hospital, 33600 Pessac, France; denis.collet@chu-bordeaux.fr; 2 Faculty of Medicine, Bordeaux Ségalen University, 33000 Bordeaux, France

## 1. Introduction

Esophageal cancer (EC) is a condition with a five-year survival rate of around 15% for all stages considered. It is the eighth most common cancer and the sixth worst prognosis because of its poor survival rate and aggressiveness [1]. It is important to distinguish the two main histological subtypes of EC with squamous cell carcinoma (SCC) and adenocarcinoma (ADC).

SCC preferentially affects the middle third of the esophagus and is associated with alcohol and tobacco abuse and a poor social setting. ADC most often affects the lower third of the esophagus and is associated with gastroesophageal reflux disease (GERD).

The prevalence of EC is currently evolving. Although SCC remains the most common cancer in the world, ADC is rapidly increasing and becoming the most prevalent form in developed countries [1].

EC’s management can be palliative or curative. Curative treatment of advanced disease requires peri-operative chemotherapy or pre-operative radiochemotherapy followed by surgery. The most frequently performed surgical procedure is esophagectomy with two-field lymphadenectomy for which the minimally invasive approach is increasingly used (MIRO trial) [2]. Advances in perioperative management and techniques have led to a decrease in postoperative morbidity and mortality. However, the improvement in long-term survival is slow. The development of targeted therapies (depending on the characteristics of the tumor) gives good hope for improving the overall prognosis. Research on the genetic characteristics of esophageal tumors and multidrug resistance are fields of study offering particularly interesting prospects. The role of palliative treatments allowing a prolonged and quality survival must also be carefully integrated in the treatment strategy.

## 2. Epidemiological Trends

### 2.1. Squamous Cell Carcinoma

Squamous cell carcinoma represents 90% of EC in the world [1]. It develops from squamous epithelium, from hyperplasia to dysplasia to cancer. At the molecular level, it is linked to a deregulation of the TP53 gene in particular.

### 2.2. Adenocarcinoma

ADC develops in Barrett’s esophagus caused by GERD. The risk factors for GERD are obesity and male gender. Two elements are responsible for the recent increase in the incidence of ADC: (i) the obesity epidemic leading to an increase in visceral adiposity and an increase in GERD, (ii) the decrease in Helicobacter pylori infection which has a protective effect on the esophagus by decreasing the production of gastric acid by increasing the pH through the production of ammonia from the urea.

## 3. The Value of Precision Imaging in EC

Imaging plays a fundamental role in the assessment of the stage and in particular for the establishment of the therapeutic strategy. The combination of different complementary imaging modalities (CT- ± PET scan and MRI) is most often used for staging. For lymph node staging, echo-endoscopy and MRI (in particular fat-suppression T2WI sequence) are the most efficient. CT and PET scan are the most interesting for the evaluation of metastases. The evaluation of the response to treatment gives valuable prognostic information [3]. The first examination performed is the upper GI endoscopy with biopsies. Current research is aimed at detecting cancer at an early stage. Thus, new approaches for early detection of cancer and surveillance of Barrett’s esophagus are being developed. The endoscopic detection of early stages of EC is difficult and this has led to the development of research in the field of artificial intelligence [4]. Deep learning has made possible the improvement of image and video processing. Prospective randomized trials are needed to confirm the value of artificial intelligence in prognosis prediction.

## 4. Medical Trends

Preneoplastic lesions and intramucosal lesions can be identified and treated by endoscopic ablation. However, in most cases, the cancer is diagnosed at an advanced stage, which is the result of extensive surgical resection more or less associated with perioperative chemotherapy or neoadjuvant radiochemotherapy. The use of neoadjuvant chemoradiotherapy improved the prognosis [5].

However, in nearly 50% of cases, patients have a metastatic disease that does not allow surgical treatment to be considered. Patients diagnosed at the metastatic stage are most often treated with palliative chemotherapy [6]. Surgical resection after chemotherapy induction may be discussed in patients with limited metastasis [7,8].

However, much progress has been made over the last few decades. These concern a better precision of the pre-therapeutic assessment, making it possible to adjust the treatment modalities as well as possible, the development of effective chemotherapies with side effects that have become acceptable, the improvement of radiotherapy techniques that make it possible to concentrate high doses in a limited volume while sparing neighborhood structures, and finally the improvement of surgical management, both at the level of perioperative care and of the surgical act itself, where the place of minimally invasive surgery is becoming clearer.

Chemotherapy, based on a combination of 5-fluorouracil, platinum salts, and taxanes, has become the standard treatment for advanced EC. The use of targeted therapies is very limited in EC and is only considered in HER 2 positive esophageal ADC [6]. Immunotherapy has transformed the management of melanoma, lung, and kidney cancers. Microsatellite instability is present in 4–20% of EC and is associated with a better prognosis than stable microsatellite tumors in association with immunosurveillance and sensitivity to immunotherapy [9].

The use of immunotherapy is currently being studied in EC. Promising results have been shown with significant improvement in overall survival in a subset of patients [10], however, not all patients benefit from immunotherapy treatment. This raises a question regarding the screening of patients who could benefit from this treatment, which depends mainly on the tumor microenvironment [11]. 

In addition, targeted therapies have been identified to have an important role in the treatment of EC targeting the epidermal growth factor receptor and the vascular endothelial growth factor. In addition, other drugs targeting surface antigens have been developed. Pembrolizumab, a PD-L1 inhibitor, has also been identified as effective for advanced SCC. These new drugs are being studied for precision medicine of EC [12]. 

## 5. Surgical Trends

Esophagectomy is the standard treatment for resectable EC more or less combined with neoadjuvant radiochemotherapy [5] or perioperative chemotherapy [13]. Perioperative docetaxel, oxaliplatin, leucovorin, and 5-fluorouracil (FLOT) chemotherapy have become the standard treatment for esophagogastric ADC [13].

However, this procedure is associated with a high risk of complications and postoperative mortality. Thus, the interest of minimally invasive surgery has gradually emerged, allowing to combine several advantages: a decrease in bleeding, post-operative morbidity, shorter hospital stay, and faster functional recovery. 

The enhanced recovery after surgery (ERAS) program in esophageal surgery is a multimodal approach that has been shown to reduce hospital stay, surgical stress and postoperative morbidity. It includes preoperative nutrition, prehabilitation, cardiorespiratory assessment, surgical and anesthetic technique, pain management, physical therapy, and early drain removal [14]. However, the concept of minimally invasive surgery is vast and is increasingly being adopted around the world [15]. Most of the published studies are heterogeneous and are monocentric retrospective studies. Thus, it can include the laparoscopic and/or thoracoscopic approach, the use of the robot. One can therefore wonder about the best surgical approach combining the lowest morbidity and mortality with optimal oncological results. Some answers can be found in randomized trials. The randomized controlled time trial compared the fully minimally invasive route to the fully open route for three-field esophagectomies indicated for cancer of the thoracic esophagus [16]. It showed a significant decrease in respiratory infections and length of hospitalization. At the same time, the number of lymph nodes harvested was no different in the two groups. 

Positive results have been shown to validate minimally invasive surgery combining thoracoscopy and laparoscopy. However, a cervical anastomosis has been performed in most cases, which is known to be associated with more pejorative outcomes [17]. One may wonder about the respective impact of laparoscopy and thoracoscopy on the reduction of post-operative outcomes. 

Indeed, laparoscopy has shown in several indications, such as cholecystectomy and anti-reflux surgery, a decrease in postoperative morbidity and a decrease in the deterioration of ventilatory mechanics. The laparoscopic approach to gastric mobilization allows reproducibility of the technique.

Thus, the Hybrid Minimally Invasive Esophagectomy for Esophageal Cancer (MIRO) randomized controlled trial compared the open route by thoracotomy and laparotomy with a hybrid route combining thoracotomy and laparoscopy for cancers of the thoracic esophagus treated by Ivor Lewis procedure [2]. A decrease in the rate of serious postoperative complications (Clavien–Dindo grades II to IV) at 30 days was shown to be 64% to 36% in the laparoscopy group (*p* < 0.0001). In addition, a decrease in pulmonary complications at 30 days of 30 to 18% was demonstrated in the laparoscopy group (*p* < 0.0001). This shows that laparoscopy, an easily reproducible technique, by itself reduces respiratory morbidity, a major complication of esophagectomy for cancer.

The Robot-Assisted Minimally Invasive Thoracolaparoscopic Esophagectomy Versus Open Transthoracic Esophagectomy for Resectable Esophageal Cancer (ROBOT) randomized controlled trial compared the open route with the totally minimally invasive robot-assisted route with manual cervical anastomosis [18]. A decrease in global and pulmonary complications was demonstrated in the robot-assisted group. The limitations of this study are explained by the fact that it was a monocentric study in an expert center and that the complication rate was particularly high in the open group.

In these three randomized trials there was no significant difference in overall long-term survival. A meta-analysis showed an 18% reduction in all-cause mortality at five years in the minimally invasive versus open group (HR 0.82; 95% CI 0.76–0.88) [19].

In addition, there was evidence of an improvement in quality of life related to a lower rate of complications [20].

To date, no randomized trial has compared the hybrid laparoscopic route to the totally minimally invasive route. Indeed, several retrospective studies have shown an increase in the rate of anastomotic fistula in the thoracoscopy group [21]. A precise study of the advantage of the thoracoscopic route and a reliable and reproducible technique are essential.

The thoracoscopic approach can be performed in lateral decubitus or prone position. The prone position would have the advantage of reducing intraoperative bleeding and, above all, avoiding one-lung ventilation and reducing post-operative pulmonary complications [22]. The major disadvantage of this approach is a conversion to a thoracotomy, which is more difficult in the case of intraoperative bleeding. To date, no randomized trial has compared the two techniques.

Other possibilities are being explored to reduce the risk of anastomotic fistula which is the main cause of postoperative mortality, such as indocyanine green [23], omental wrapping [24] and pleural tenting [25].

A new technique proposing a hybrid endovascular and surgical preconditioning of the stomach has shown interesting results in a porcine model [26]. Indeed, anastomotic complications are supposed to be related to insufficient perfusion of the gastric transplant. Hybrid preconditioning improves the perfusion of the gastric transplant and potentially reduces postoperative complications. These preliminary results need to be confirmed in humans. Although the morbi-mortality of esophagectomies remains one of the highest in gastrointestinal surgery, it has drastically decreased over the last few decades, allowing surgery to confirm its place in the treatment of EC and to once again be a leading treatment for resectable EC. To this should be added the tendency to group together this pathology in centers specialized in high-volume activity.

Therapeutic advances are making it possible to manage increasingly older patients. Data on elderly patients are sparse because older patients are rarely included in clinical trials.

Patients over 60 years of age were significantly affected. In order to determine the best treatment option, it is important to assess the patient’s co-morbidities. Although surgical resection can be considered with or without neoadjuvant treatment, exclusive radiochemotherapy may be an interesting therapeutic alternative.

## 6. Genetics Advances in EC

EC is associated with mutations in *TP53*, *NOTCH* and *MTOR* genes. ADC is often associated with HER2-Neu overexpression. Individual variations in the prognosis of EC point to the need for advances in personalized therapy. In a systematic review of the literature, gene expression profiles were linked to survival in nine studies [27].

Long non-coding RNA and microRNA are significant regulators of gene expression and chromatin configuration and have a key role in esophageal carcinogenesis and may alter the response to some targeted therapies. Several long non-coding RNA and miRNA panels have been reported to be associated with survival of EC patients and may be prognostic markers, especially in the evaluation of response to therapy [28].

Circular RNA, an endogenous non -coding RNA with a circular structure can link specifically to miRNA and directly or indirectly regulate gene expression in EC and could also be used as biomarkers [29].

## 7. Multidrug Resistance

Multidrug resistance (MDR) is a major obstacle to effective treatment, particularly in recurrent tumors. Several approaches are being investigated to understand the mechanisms of resistance to therapeutics in EC. First, the plasticity of cancer stem cells has been identified as playing a role in treatment resistance in relation to their heterogeneity. Second, their key signaling pathways (Wnt/β-catenin, Notch, Hedgehog, YAP, JAK/STAT3) modulate CSCs during EC progression [30].

The exploration of resistance mechanisms has highlighted the DNA damage response, epithelial mesenchymal transition, metabolic reprogramming, and the interaction between CSCs and their environment. Thus, the targeting of CSCs could open the way to new therapeutic strategies for EC. Moreover, epigenetic deregulation and the hypoxic microenvironment have been shown to play a role in resistance to radiotherapy [31].

Recent randomized trials have demonstrated the value of anti-PD1 and anti-PDL1 in the treatment of advanced or metastatic EC [32,33] and as adjuvant treatment after R0 resection [34]. 

Current research perspectives on EC are based on both multiomic characterization and validation of cancer aggressiveness elements in order to establish an atlas of tumor aggressiveness and guide future treatment strategies, and also on targeting cancer stem cells [35].

Circulating tumor cells (CTCs) have been identified as new biomarkers, providing very important clinical information to predict cancer prognosis, monitor therapeutic response or the mechanism of metastasis of different cancers. The isolation of CTCs is an application of liquid biopsy.

A recent meta-analysis confirmed the diagnostic value of liquid biopsies using a molecular combination and EC and showed that the presence of CTCs is associated with a poor prognosis [36]. Liquid biopsies in EC are a particularly interesting area of research for the future.

## 8. Palliative Care

Palliative management has taken a major place in the management of advanced EC, particularly dysphagia, the management of which can improve quality of life and nutritional status [37].

## 9. Conclusions

Over the last few years, we have witnessed an evolution in the epidemiology of EC, a medical treatment with a particular focus on targeted therapies, and surgical treatment with the predominance of the minimally invasive approach. The objective of this special issue is to provide an overview of the current management of EC concerning pre-treatment assessment, perioperative treatment, nutritional management, surgical treatment, and special situations. The ambition is to be an aid in the daily practice of physicians confronted with EC.
